# Biological scaling in green algae: the role of cell size and geometry

**DOI:** 10.1038/s41598-021-93816-2

**Published:** 2021-07-13

**Authors:** Helena Bestová, Jules Segrestin, Klaus von Schwartzenberg, Pavel Škaloud, Thomas Lenormand, Cyrille Violle

**Affiliations:** 1 Department of Botany, Faculty of Science, Charles University, Benátská 2, Prague, Czech Republic; 2 CEFE, Univ Montpellier, CNRS, EPHE, IRD, 1919 Route de Mende, 34293 Montpellier, France; 3 Microalgae and Zygnematophyceae Collection Hamburg, Institute of Plant Science and Microbiology, Universität Hamburg, Ohnhorststr. 18, 22609 Hamburg, Germany

**Keywords:** Natural variation in plants, Plant physiology

## Abstract

The Metabolic Scaling Theory (MST), hypothesizes limitations of resource-transport networks in organisms and predicts their optimization into fractal-like structures. As a result, the relationship between population growth rate and body size should follow a cross-species universal quarter-power scaling. However, the universality of metabolic scaling has been challenged, particularly across transitions from bacteria to protists to multicellulars. The population growth rate of unicellulars should be constrained by external diffusion, ruling nutrient uptake, and internal diffusion, operating nutrient distribution. Both constraints intensify with increasing size possibly leading to shifting in the scaling exponent. We focused on unicellular algae *Micrasterias*. Large size and fractal-like morphology make this species a transitional group between unicellular and multicellular organisms in the evolution of allometry. We tested MST predictions using measurements of growth rate, size, and morphology-related traits. We showed that growth scaling of *Micrasterias* follows MST predictions, reflecting constraints by internal diffusion transport. Cell fractality and density decrease led to a proportional increase in surface area with body mass relaxing external constraints. Complex allometric optimization enables to maintain quarter-power scaling of population growth rate even with a large unicellular plan. Overall, our findings support fractality as a key factor in the evolution of biological scaling.

## Introduction

The Metabolic Scaling Theory (MST) states that the pace of organismal processes, is tightly linked to body size (M)^1^, following power-law functions such as:

1 $$Y = Y_{0} M^{b}$$

where *Y* represents metabolic rate or growth rate, *M* organismal body mass, *Y*_0_ a normalization constant and *b* the scaling exponent. MST predicts *b* to be a multiple of 1/4, precisely 3/4 for metabolic rate^2–4^. As metabolism sustains biomass production for growth and reproduction, organismal allometry relationships can be extended to population growth rate^1,5^, The MST hypothesis underlying the scaling from individual energetics (and its drivers, including body size) to population growth rate are that: (i) population growth is fueled by the acquisition and allocation of energy, and (ii) the acquisition and allocation of energy are constrained by body size (and shape, i.e., the fractal resource distribution networks). Thus, MST offers a metabolic explanation for the scaling relationship of population growth to body size^1^. According to Eq. (1), mass-specific production rates and mortality rates scale with body size with an allometric coefficient equal to − 1/4 (*b* − 1)^1^. Consequently, population growth rate (*µ*_*max*_) should scale with body size with the same allometric coefficient of − 1/4^1,6–8^. The quarter-power allometric scaling, also known as Kleiber’s law, contradicts simple “surface area law” where *b* is a multiple 1/3, reflecting the outcome of the geometric scaling between volume and surface area^9^. Based on hypothesis that allometric relationships reflect optimal phenotype resulting from natural selection and constrained by physical laws^10^, a universal metabolic scaling across a wide range of organisms would therefore indicate the existence of common biophysical constraints and optimal body plan. However, DeLong et al.^5^ highlighted changes in the allometric scaling of metabolic rate and *µ*_*max*_ along main evolutionary transitions, linked with “innovations in metabolic design”, such as cell compartmentalization and multicellularity. Their meta-analysis validated Kleiber’s law for metazoans only, whereas bacteria and protists deviated from the − 1/4 exponent. Kempes et al.^11^ also showed variation in population growth rate across taxa using a model partitioning metabolic costs between biosynthesis and maintenance. These findings challenged the universality of Kleiber’s law and the existence of a common metabolic constraint for all organisms^12^. The general agreement on the form and the cause of growth rate-size relationship is still lacking, notably when navigating across the tree of life.

In protists, two types of physical constraints can regulate cell metabolism. First, external constraints govern uptake of resources from external milieu. They are linked to cell surface area that limits the pace of diffusion and the number of transport sites at the cell membrane^5,13^. Second, internal diffusion constraints, distances and viscosity within the cytoplasm in particular, control the distribution of resources within the cell and are related to cell volume, mass, and shape^14^. The ability of intracellular diffusion to maintain a constant concentration of solutes^15^ and CO_2_^16^ is known to decrease with size. At the upper limit of protist size, external and internal constraints should lead to reduced metabolic efficiency (a shift in metabolic scaling) and competitive superiority of multicellular organisms^5^. Nevertheless, both constraints could be minimized by body plan optimizations, especially by changing body morphology and diluting cell content^14,17^. Those changes in body plan certainly increase surface to active cell volume ratio^9,17^, but it is little known to what extent such allometric adjustments influence the scaling of organismal processes.

**Figure 1:**
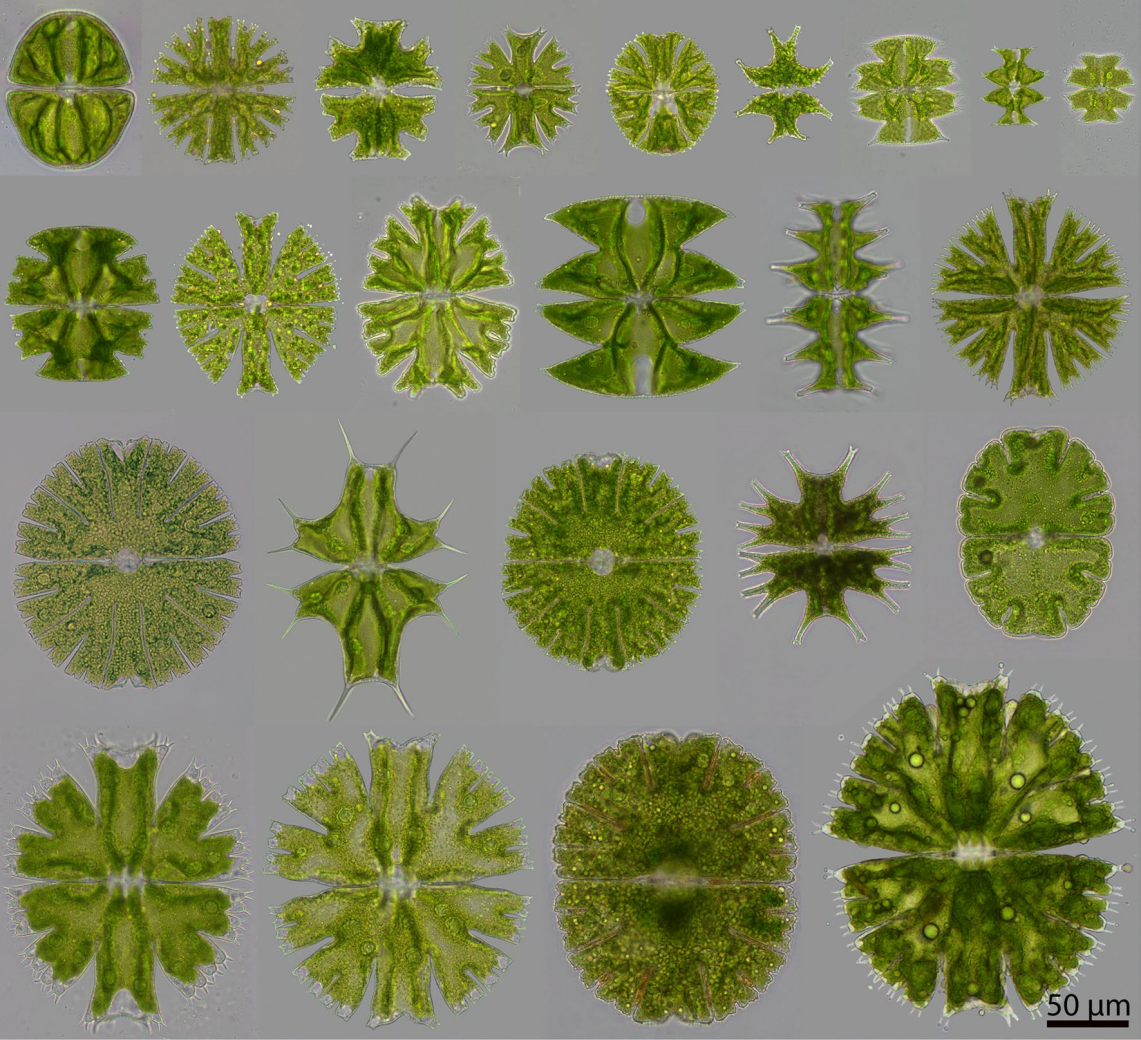
Size and morphological variability in *Micrasterias*. Microphotographs of 24 strains used in the experiment. From right to left: first line *M. ralfsii, M. radiosa, M. crux-melitensis, M.radians, M. conferta, M. tropica, M. ceylanica, M. pinnatifida, M. decemdentata*; second line: *M. truncata, M. novae-terrae, M. americana, M.laticeps, M. muricata, M. papilifera, M.thomasiana (K602), M. ceratofera, M. thomasiana (SCVK 8), M. furcata, M. jenneri,* third line: *M. apiculata, M. rotata, M. denticulata, M. fimbriata*.

In multicellular organisms, the fractal resource transportation networks were proposed as an optimization strategy that overcomes internal distribution constraints and leads to optimal quarter-power scaling^4^. The fractal networks are typically missing in unicellular organisms. However star-shaped morphology of streptophyte green algal genus *Micrasterias* (Streptophyta, Desmidiales, Zygnematophyceae) strikingly resemble them. The extraordinary diversity of sizes and shapes within a single genus (Fig. 1) and among species with a similar ecology offers a unique opportunity to examine the role of body plan optimization, especially fractality. Neustupa^18^ reported elevated surface-area to volume allometry of *Micrasterias* and showed that cell branching compensates for large cell volume. It therefore reduces biophysical constraints, potentially affecting the rate of organismal processes (e.g. *µ*_*max*_), however relationship between morphology and population growth rate remains unexplored in unicellular organisms. In this study, we carried out an experiment investigating growth allometries of 24 species of *Micrasterias* displaying a large range of sizes (spanning two orders of magnitude) and morphological complexity (Fig. 1).

We experimentally measured population growth rate and analysed its size dependency using size measurements (volume and body mass). To understand the constraints that govern the observed allometric relationships we also measured morphological traits related to body plan optimization. Specifically, we quantified the increase in surface area due to fractality and flattening of the cell. We investigated the role of morphology, more precisely the effect of body plan optimization on growth rate scaling and shifts in surface and body mass allometries.

## Results

**Figure 2:**
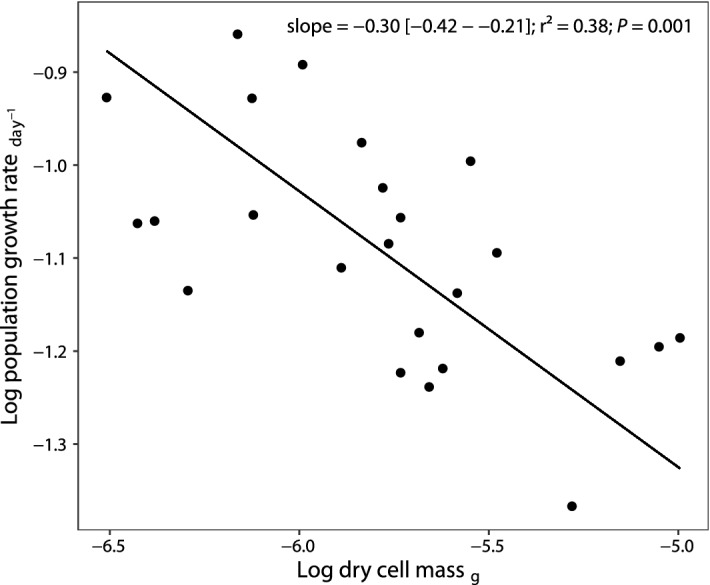
Population growth rate as a function of dry cell mass for the 24 studied species of Micrasterias. Both variables are log-10-transformed. Each dot represents the average value of a given species. The black line represents SMA regression.

We found that the scaling of population growth rate with body mass followed Kleiber’s law. The slope of the standardized major axis regression (SMA)^19^ between *µ*_*max*_ and body mass (dry cell mass) was − 0.30 (Fig. 2, Table 1) without differing from − 0.25 (test for SMA slope different from − 0.25: *P* value = 0.3). The slope between *µ*_*max*_ and volume displayed a flatter slope − 0.23. All allometric relationships are summarized in Table 1.

**Table 1:** Standardized major axis (SMA) regression of population growth rate and traits linked to body size.

Response	Predictor	Slope	[95% CI interval]	Intercept	*r*^2^	*P* value
Growth rate	Dry cell mass	− 0.30	[− 0.42, − 0.21]	− 2.81	0.38	0.001
	Cell volume	− 0.23	[− 0.31, − 0.17]	0.07	0.48	< 0.001
	Cell surface	− 0.26	[− 0.38, − 0.18]	0.16	0.26	0.01
Dry cell mass	Cell volume	0.77	[0.62, 0.96]	− 9.72	0.76	< 0.001
Cell surface	Cell volume	0.87	[0.69, 0.96]	0.33	0.72	< 0.001
	Dry cell mass	1.12	[0.87, 1.45]	11.27	0.65	< 0.001

All variables are log-10-transformed. *P* value is test of slope (correlation coefficient) against 0.

Size-related traits (dry cell mass, volume and surface) were negatively correlated with *µ*_*max*_ (Table 1). On the other hand, neither size (volume and dry cell mass) nor *µ*_*max*_ were correlated with traits associated with cell shape such as departure from circular outline—*circularity (α)*, relative surface gain by cell lobulation—*degree of fractalization (β)* and by flattening—*degree of flattening (γ)*, and absolute difference of surface of the cell and surface of a sphere of equal volume—*gain in surface (δ)* (see Table of correlations S3 in Supplementary information—Appendix 1)*.* Cell density, expressed as dry cell mass-to-volume ratio, was positively correlated with *µ*_*max*_ (Pearson’s r = 0.44, *P* = 0.03, Table S3), but, was not correlated to the residuals of the *µ*_*max*_ versus dry cell mass scaling (Pearson’s r = 0.21, *P* = 0.33, Table S4 in Supplementary information—Appendix 1). Similarly, no correlation was found between the residuals of the *µ*_*max*_ versus dry cell mass and cell circularity (α), degree of fractalization (β), degree of flattening (γ), overall gain in surface (δ) nor cell surface area (Table S4 in Supplementary information—Appendix 1). Therefore, higher surface area at a given cell size did not result in higher population growth rate. The correlation of residuals from *µ*_*max*_ versus cell volume is given in Table S4.

**Figure 3:**
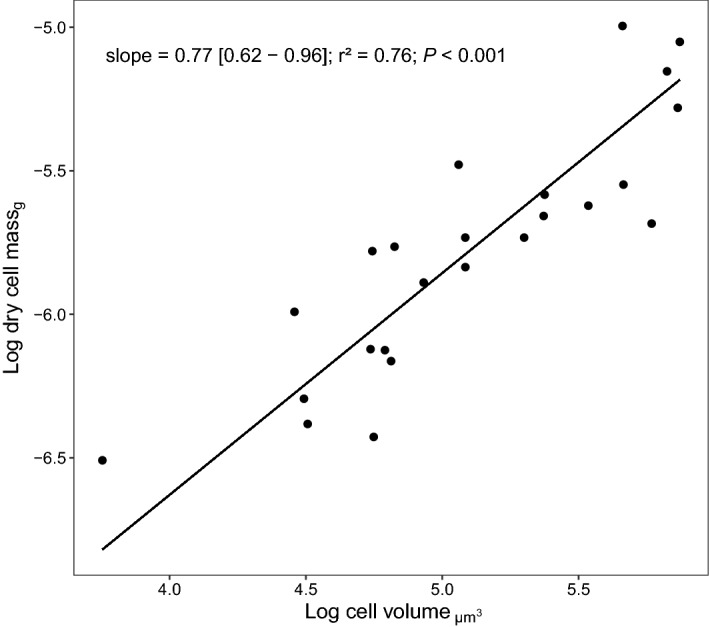
Sublinear scaling of dry cell mass to volume in 24 species of *Micrasterias*. Both variables are log-10-transformed. Each dot represents the average value of a given species. The black line represents SMA regression.

**Figure 4:**
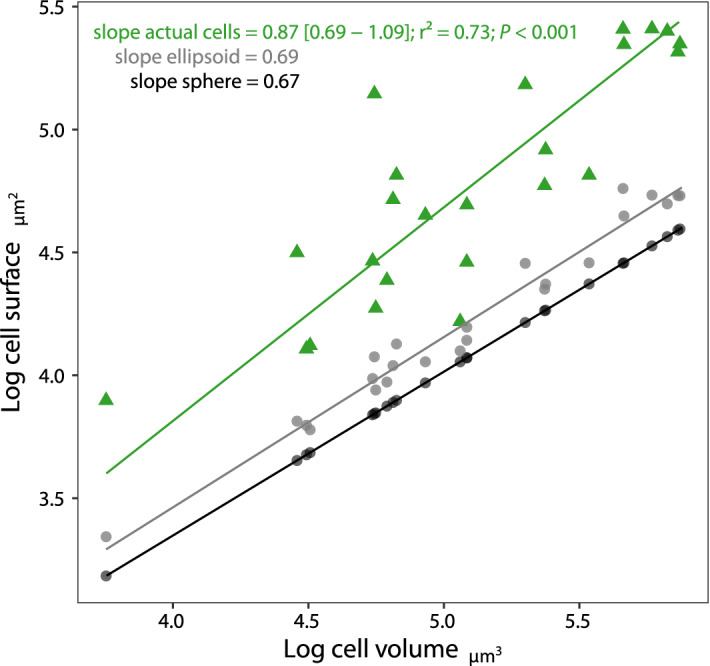
Comparison of surface area to volume scaling of *Micrasterias* cells (green triangles) and modelled theoretical shapes. Grey points represent spheres with a volume equal to actual cells. Black points represent ellipsoids with equal volume and same axis proportions (i.e. same global flattening). Only actual cells that are both flattened and with fractal-like morphology depart from Euclidean scaling (2/3). Lines represent SMA regressions and variables are log10-transformed. ***P value < 0.001.

We found that body mass scaled sublinearly with cell volume (SMA slope = 0.77; CI = [0.62–0.96]) (Fig. 3, Table 1). Scaling of cell surface area to volume significantly departed from Euclidean scaling of 2/3 (Fig. 4; Table 1; SMA slope = 0.87; CI = [0.69–1.09]). Cell flattening and fractality both led to an increase in surface area, compared to modelled theoretical shapes with equal volumes, ellipsoid without lobulation and sphere without flattening and lobulation (Fig. 4). However, the slope of the surface to volume scaling for an ellipsoid did not differ from 2/3 (SMA slope = 0.69; CI = [0.64–0.75]). Only fractality elevated the slope of the surface to volume scaling from 2/3 (test for SMA slope different from 0.6667: *P* value = 0.024). Moreover, surface area increased isometrically with body mass (Table 1, SMA slope = 1.12; CI = [0.87–1.45]). Residuals from surface area to volume scaling were negatively correlated with departure from cell circularity (Pearson’s *r* =  − 0.73, *P* value < 0.001), positively with degree of fractalization (Pearson’s *r* = 0.86, *P* value < 0.001) and degree of flattening (Pearson’s *r* = 0.74, *P* value < 0.001).

## Discussion

*Micrasterias* possibly represents a key transitional group in the evolution of allometry, offering a unique opportunity to investigate geometrical constraints of cell morphology on growth rates. We measured the growth rate, size, and morphological traits of 24 *Micrasterias* species. DeLong et al.^5^ predicted that large unicellular organisms would have lower mass specific metabolic rates, due to increased external and internal constraints. We found that size-dependent variation in maximum population growth rate (*µ*_*max*_) of *Micrasterias* did not depart from Kleiber’s law^20^, implying existence of mechanisms that lessen such constraints.

The most apparent surface area optimization strategies in *Micrasterias* are cell fractality and flattening. West et al.^4^ proposed fractality as a feature elevating scaling slope of surface area to volume of resource-transport networks to allometrically optimal 3/4. Our results support the hypothesis that fractality in *Micrasterias* arose as a mechanism elevating the scaling of the external surface area^18^. Indeed, the positive surface to volume allometry (*b* = 0.87) was essentially caused by fractality and was not statistically different from the “optimal” 3/4 value (see Fig. 4). Flattening increased surface area but without departure from isometry of 2/3, contrary to the model developed by Okie^17^, where planar (flattened) cell have higher surface area volume scaling. Moreover, residual variation from the surface to volume scaling was positively correlated with fractality. Species with a higher degree of fractality and larger departure from circular shape had an even larger increase in surface area. Interestingly, Neustupa^18^ reported similar values of surface area to volume scaling (*b* = 0.91) for a different set of *Micrasterias* species. Yet, our results showed that traits linked to cell shape (fractality, flattening, circularity, and overall gain in surface area) were not correlated with *µ*_*max*_ nor with species-specific deviation from the general Kleiber’s law (residuals from *µ*_*max*_ body mass relationship). Despite the fact that morphological adaptations cannot be directly linked with population growth rate, they still could be crucial for relaxing biophysical constraints, and could be at the origin of quarter-power scaling of relatively large cells of *Micrasterias*. The outer body fractality of *Micrasterias* differs from the self-similar organization of resource-transport networks proposed by West et al.^3,4^, but it functions similarly. It maximizes the exchange surface area to the volume needed to be supplied. Open question remains, whether it possibly could also represent a branching distributional network facilitating inner diffusion transport. The shape and its link to external diffusion have already been described^21,22^, but the role of cell shape on internal diffusion is often neglected. In spherical cells CO_2_ concentration rapidly declines towards the centre^16^. Change in cell morphology, eg. elongation, would reduce the average distance to membrane and therefore minimize the resource limitation in central parts of cell^14,16^. Hence, the flat and fractal morphology of *Micrasterias* partially lessens intracellular diffusion constraint, but a distribution of nuclear transcripts to extremities would still likely limit the maximal cell size^14,15,23^. Furthermore, the shape of organisms undoubtedly affects the distribution of organelles within the cell. This can lead to heterogeneous use of resources within the cell. However, the effect of cell shape on metabolism is rarely considered and should be further explored.

The cell size has two components: volume and mass. Our results indicate that fractality is not the only mechanism to alleviate size-related constraints. Cell mass scaled with volume sublinearly (*b* = 0.77). Sublinear scaling of mass with volume is not new^9,14,24^, but it is particularly noteworthy. Many metabolic scaling studies assume size-invariant density^5,25^. However, cell density can be reduced either by increasing the proportion of vacuoles^17^ or by decreasing molecular crowding^14^. Such dilution of cell content is an important allometric optimization strategy^17^. The dilution enables an increase in size without a major increase in metabolic active volume, it decreases phosphorus and nitrogen minimal quotas^26^ and leads to dilution of metabolic and structural components^17^. An experimental test done by Gallet et al.^14^ showed that the evolution of bigger and faster-growing bacteria is linked to cell dilution. However, here we did not find similar results since cell density was not related to *µ*_*max*_ or its residuals.

What are the biophysical constraints underlying − 1/4 scaling in *Micrasterias*? Under influence of external constraint limitations we would expect that increase in surface area would lead to increase in population growth rate_*.*_ However, we found negative scaling of *µ*_*max*_ with the surface area (*b* =  − 0.26) (Table 1) and no correlation between surface area and residuals from *µ*_*max*_ body mass relationship. On the other hand, the negative quarter-power relationship between *µ*_*max*_ and body mass is consistent with resource-transport models, where the *µ*_*max*_ limitation is caused by constraints associated with nutrient transport^3,25^. Our results therefore suggest that *µ*_*max*_ scaling is mostly governed by internal constraints, in agreement with an experiment of Marañón et al.^27^ on phytoplankton. The value of the scaling exponent they reported for cells larger than 40 µm^3^ did not differ from the value observed in our experiment. Marañón et al.^27^ argued that nutrient uptake abilities of larger cells are higher than their requirements, therefore the growth of larger cells are likely to be limited by internal transport and assimilation only. The intracellular diffusion limitation is often overlooked but could represent a very important constraint in unicellular organisms^14^. It is also a good candidate for a constraint limiting the upper size of the unicellular body^23^. It would explain why extremely large protists, such as *Halimeda* or *Caulerpa*, have siphonous body plans (large cell with multiple nuclei and cytoplasm streaming). Multinucleate cells would minimize the limiting distance from the nucleus to the periphery^15^. Additionally, active transport of metabolites within cell facilitated by cytoplasm streaming would be also crucial to overcome internal diffusion constraints in very large cells^15^.

In conclusion, even though surface area to volume ratio is typically less favourable for large sizes^28^, our data showed that, population growth rate of *Micrasterias* is likely not limited by surface area. The geometric adaptations of *Micrasterias* altered the scaling between surface area and mass. Overall, we highlighted that complex morphological diversification strategies can represent “evolutionary escape route from constraints imposed on physiological functions”^28^. Combination of size-related shape modification and cell dilution might enable large unicellular organisms not to be limited by surface area and maintain quarter-power scaling of population growth rate.

## Methods

We used monocultures of 24 strains of the genus *Micrasterias* (Fig. 1) for our study. Used strains are available in public culture collections MZCH-SCVK Hamburg Collection^29^ and CAUP Culture Collection Prague^30^. Details about strains used can be found in Supplementary information—Appendix 1 (Table S1).

### Assessment of species growth rate

We transferred storage cultures to fresh DY IV modified medium with pH 5.7 (botany.natur.cuni.cz/algo/caup.html), at a constant temperature of 20 °C and with constant illumination. After 9 days of accommodation, we inoculated four replicates per strain on 12-well plates to a starting concentration of 100 cells mL^−1^ with the total volume of 4 mL. We sealed the well plates with the Breathe-Easy sealing membrane to prevent evaporation. Every four days we sampled 10% of volume (400 µL) and replaced it with equal volume of fresh medium. We preserved samples with formaldehyde (1% final concentration) and later used them to estimate cell abundances We followed the growth of strains for 35 days to cover the exponential phase of growth.

We estimated cell abundance in samples using Nageotte hemacytometer (Bright Line, Hausser Scientific, Horsham, PA). We modelled growth rates by fitting cubic smoothing spline to natural logarithm of cell abundance versus time in days. Smoothing spline produces more accurate estimates of growth parameters than parametric fits^31^. We estimated the maximum population growth rate (*µ*_*max*_) as the first derivate of the smoothing spline using the package *growthrates* ver. 0.7.2.^32^. By fitting growth curve individually to each replicate, we were able to identify maximum population growth rate, before density-dependent mechanisms may have slowed down the reproduction. We calculated the final growth rate for each strain as the maximum population growth rate averaged across replicates.

### Measurements of cell volume and cell surface area

**Figure 5:**
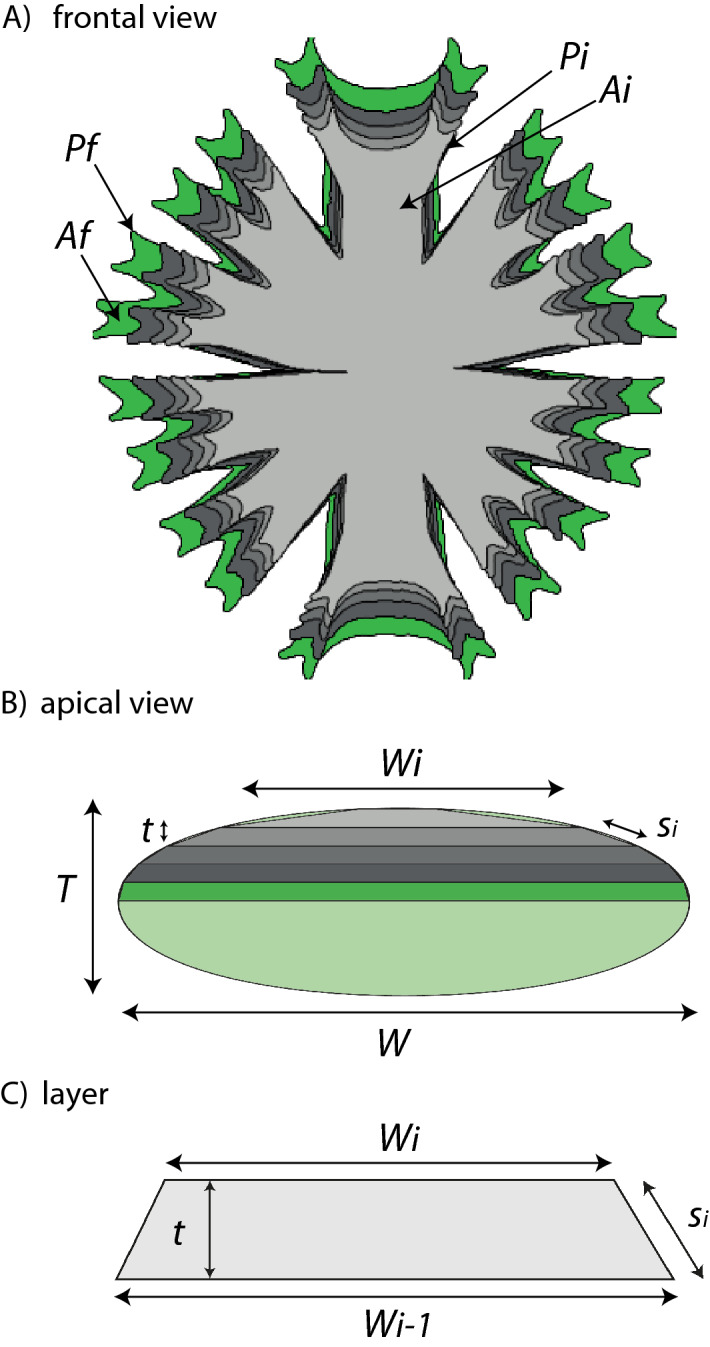
Illustration of a 3D mathematical model of cell created by stacking cross-sectional layers. (**A**) frontal view, green layer is the frontal view of the cell (**B**) apical view of stacked layers, green outline represents the apical view of the cell. Frontal view of the cell represents the biggest slice with the area ($A_{f}$) and the perimeter ($P_{f}$). Apical view of the cell defined maximal cell width ($W$) and the thickness ($T$). (**C**) Stacked layers were of equal thickness ($t$) and we measured width ($W_{i}$) from which we calculated perimeter ($P_{i}$), area ($A_{i}$), and slant height $\left( {s_{i} } \right)$, distance measured on lateral side.

Estimating volumes and surface areas of cells with complex shapes is not an easy task. Commonly cell shape is assigned to general geometrical shape, and volume is calculated based on the mathematical formula for this geometrical shape^33^. This approach would lead to inaccuracies for morphologically complex cells like *Micrasterias*. Previously, other method optimizing volume and surface area of more complex cells been developed^34^. However, we believe, that our approach refines the measurement as it allows to account for both complex frontal and apical morphology. We created a 3D model by dividing the shape into flat cross-sectional layers, each layer represented by a frustrum – part of a solid between two parallel planes (Fig. 5).

These layers are of known thickness, area, and perimeter. When layers are thin and numerous, this model accurately approximates the volume and surface area of the real object. Therefore, several cell dimensions were measured on frontal and apical views of *Micrasterias* cells made by an Olympus BX51 microscope (Olympus Tokyo Japan) associated with a Canon EOS 60D. We performed image analysis by pixel counts on binary images in software Fiji^35^ and R Statistical Software^36^.

The frontal view represents the central and the largest layer of the cell. We measured the area ($A_{f}$) and the perimeter ($P_{f}$) of the frontal view (Fig. 5). The apical view defines the maximum cell width ($W$) and the cell thickness ($T$). The picture was divided into *n* slices of equal thickness $t = \frac{T}{n}$. For each slice $i$, we measured the maximum slice width ($W_{i}$) from the apical cross section. We calculated $f_{i}$ defining the ratio between slice width $W_{i}$ and the maximum width of the cell width $W$ as $f_{i} = \frac{{W_{i} }}{W}$ . For each slice *i*, the frontal area is therefore $A_{i} = A_{f} f_{i} ^{2}$ and the perimeter $P_{i} = P_{f} f_{i}$. Subsequently the volume and area of lateral side of each layer can be calculated using formulas for frustrum, volume as $V_{i} = \frac{t}{3}\left( {A_{i} + A_{{i + 1}} + \sqrt {A_{i} + A_{{i + 1}} } } \right)$ and area of the lateral side as $S_{i} = \frac{1}{2}s_{i} \left( {P_{i} + P_{{i + 1}} } \right)$, where $s_{i}$ is the slant height $s_{i} = \sqrt {t^{2} + \left( {\frac{{W_{i} - W_{{i + 1}} }}{2}} \right)^{2} }$. The total volume ($V$) of the cell is approximated by summing over the slices $V = \sum\nolimits_{{i = 1}}^{n} {V_{i} }$. Similarly, the total surface area ($S$) of the cell is approximated by the sum of each slice lateral area ($S_{i}$) and twice the area of smallest slice ($A_{n}$): $S = 2A_{n} + \sum\nolimits_{{i = 1}}^{n} {Si}$. Additional information about surface area and volume calculation together with test of calculations on spheres of different radius, can be found in Supplementary information—Appendix 2.

We measured frontal dimensions of 50 cells from the same culture. We used one cell to define the apical outline and ratio between cell width and thickness. Based on this we calculated the thickness of measured cells. The final surface area and volume are the mean values of 50 cells per species.

### Geometrical summaries of cell shapes

We computed different indices to summarize the cell shapes. First, we used circularity:

2 $$\alpha = 4\pi A_{f} /P_{f}^{2}$$

to measure the departure from a circular outline for the frontal face. This index ranges from 1 for a perfect circle to 0 for an infinitely elongated polygon^37^. Second, we computed the degree of fractalization:

3 $$\beta = \frac{S}{{S_{{ellipsoid}} }}$$

to measure the gain of surface area provided by cell branching. To do so, we computed the ratio of the actual cell surface area ($S$) to the surface area ($S_{{ellipsoid}}$) of an ellipsoid with the same volume and axes proportion as the observed cell. The three axes of the observed cell are the length ($L$), width $\left( W \right)$ and thickness ($T$). To compute $S_{{ellipsoid}}$, we first estimated how much length of these axes need to be shrunk to keep the volume equal to the observed volume, by finding the coefficient *k* so that $V = \frac{4}{3}\pi \times kL \times kW \times kT$. Then we measured the surface area of the resulting ellipsoid using Knud Thomsen's approximation, $S_{{ellipsoid}} \approx 4\pi \sqrt[p]{{\frac{{a^{p} b^{p} + a^{p} c^{p} + b^{p} c^{p} }}{3}}}$ where *p* ≈ 1.6075, and where *a*, *b* and *c* are axis lengths^38^ corresponding to *kL*, *kW* and *kT*.

Third, we used:

4 $$\gamma = \frac{{S_{{ellipsoid}} }}{{S_{{sphere}} }}$$

to measure the degree of cell flattening. This index indicates the surface area gain obtained by cell flattening in absence of any lobulation or fractalization. To compute $S_{{sphere}}$, we simply computed the surface area of the sphere that would have the same volume as the observed cell. Finally, we computed the overall gain in surface area compared to a sphere with equal volume as:

5 $$\delta = \beta \gamma = \frac{S}{{S_{{sphere}} }}.$$

### Measurement of cell mass

We measured cell mass from a dried weight of storage cultures grown under experimental conditions. We centrifuged four replicates of 2 mL from storage cultures at 1500 rpm for ten minutes using an Eppendorf 5424 centrifuge. We resuspended pellets in a small amount of fresh medium (approximately 200 µL), transferred the suspension into previously weighted pressed tin capsules and dried those 48 h at 70 °C. We placed the capsules in a desiccator to cool down at room temperature and weighted those using a Sartorius MC5 microbalance. We estimated cell weight by dividing the dry weight of pellets by the number of cells in 2 mL, previously estimated in storage cultures using a Nageotte hemacytometer. We calculated cell density as dry mass-to-volume ratio.

### Statistical analysis

We used standardized major axis regression (SMA)^19^ to estimate the scaling relationships between traits, all traits were transformed by common logarithm (base 10). SMA regressions were computed using the *smatr* R-package ver. 3.4-3^39^. To account for phylogenetic effects, we performed phylogenetic standardized major axis regression using the R-package *phytools* ver. 0.6-60^40^. For the latter, we estimated the lambda parameter by maximum likelihood. We constructed phylogenetic covariance matrix using published multi-loci phylogeny of *Micrasterias*^41^. Slopes of phylogenetic SMA did not differ from SMA slopes (see Table S2 in Supplementary information—Appendix 1). Therefore, we further present only the results from SMA regression and take all the relationships as phylogenetically independent. We tested the correlations between traits and between SMA residuals of *µ*_*max*_ scaling and morphological and size-related traits in order to describe the influence of allometric optimization on departure from this scaling.

All statistical analyses were performed using R Statistical Software^36^.

## Supplementary Information

Supplementary Information.

## References

[1] Brown JH, Gillooly JF, Allen AP, Savage VM, West GB (2004). Toward a metabolic theory of ecology. Ecology.

[2] Savage VM, Deeds EJ, Fontana W (2008). Sizing up allometric scaling theory. PLoS Comput. Biol..

[3] West GB, Brown JH, Enquist BJ (1997). A general model for the origin of allometric scaling laws in biology. Science.

[4] West GB, Enquist BJ, Brown JH (1999). The fourth dimension of life: Fractal geometry and allometric scaling of organisms. Science.

[5] DeLong JP, Okie JG, Moses ME, Sibly RM, Brown JH (2010). Shifts in metabolic scaling, production, and efficiency across major evolutionary transitions of life. Proc. Natl. Acad. Sci. U. S. A..

[6] Blueweiss L (1978). Relationships between body size and some life history parametrs. Oecologia.

[7] Fenchel T (1974). Intrinsic rate of natural increase: The relationship with body size. Oecologia.

[8] Savage VM (2004). The predominance of quarter-power scaling in biology. Funct. Ecol..

[9] Niklas KJ (1994). Plant Allometry: The Scaling of form and Process.

[10] Enquist BJ, Tiffney BH, Niklas KJ (2007). Metabolic scaling and the evolutionary dynamics of plant size, form, and diversity: Toward a synthesis of ecology, evolution, and paleontology. Int. J. Plant Sci..

[11] Kempes C, Dutkiewicz S, Follows MJ (2012). Growth, metabolic partitioning, and the size of microorganisms. Proc. Natl. Acad. Sci. U. S. A..

[12] West GB, Brown JH (2005). The origin of allometric scaling laws in biology from genomes to ecosystems: towards a quantitative unifying theory of biological structure and organization. J. Exp. Biol..

[13] Yoshiyama K, Klausmeier CA (2008). Optimal cell size for resource uptake in fluids: A new facet of resource competition. Am. Nat..

[14] Gallet R (2017). The evolution of bacterial cell size: the internal diffusion-constraint hypothesis. ISME J..

[15] Beardall J (2009). Allometry and stoichiometry of unicellular, colonial and multicellular phytoplankton. New Phytol..

[16] Wirtz KW (2011). Non-uniform scaling in phytoplankton growth rate due to intracellular light and CO 2 decline. J. Plankton Res..

[17] Okie JG (2013). General models for the spectra of surface area scaling strategies of cells and organisms: fractality, geometric dissimilitude, and internalization. Am. Nat..

[18] Neustupa J (2015). Static allometry of unicellular green algae: Scaling of cellular surface area and volume in the genus Micrasterias (Desmidiales). J. Evol. Biol..

[19] Warton DI, Wright IJ, Falster DS, Westoby M (2006). Bivariate line-fitting methods for allometry. Biol. Rev. Camb. Philos. Soc..

[20] Savage VM, Gillooly JF, Brown JH, Charnov EL, West GB (2004). Effects of body size and temperature on population growth. Am. Nat..

[21] Karp-Boss L, Boss E, Kana TM, Glibert PM (2016). The elongated, the squat and the spherical: selective pressures for phytoplankton shape. Aquatic Microbial Ecology and Biogeochemistry: A Dual Perspective.

[22] Young KD (2006). The selective value of bacterial shape. Microbiol. Mol. Biol. Rev..

[23] Ryabov A (2021). Shape matters: the relationship between cell geometry and diversity in phytoplankton. Ecol. Lett..

[24] Menden-Deuer S, Lessard EJ (2000). Carbon to volume relationships for dinoflagellates, diatoms, and other protist plankton. Limnol. Oceanogr..

[25] Banavar JR (2010). A general basis for quarter-power scaling in animals. Proc. Natl. Acad. Sci. U. S. A..

[26] Niklas KJ (1994). Size-dependent variations in plant growth rates and the ‘3/4-power rule’. Am. J. Bot..

[27] Marañón E (2013). Unimodal size scaling of phytoplankton growth and the size dependence of nutrient uptake and use. Ecol. Lett..

[28] Niklas KJ, Cobb ED (2017). Size-dependent variation in plant form. Curr. Biol..

[29] Schwartzenberg VK, Bornfleth S, Linder AC, Hanelt D (2013). The Microalgae and Zygnematophyceae Collection Hamburg (MZCH)—living cultures for research on rare streptophytic algae. Arch. Hydrobiol. Suppl. Algol. Stud..

[30] Škaloud P, Neustupa J (2009). CAUP—The Culture Collection of Algae of Charles University in Prague.

[31] Kahm M, Hasenbrink G, Ludwig J, Lichtenberg-Fraté H, Kschischo M (2010). grofit: Fitting biological growth curves with R. J. Stat. Softw..

[32] Petzoldt, T. Growthrates: Estimate Growth Rates from Experimental Data. R package version 0.7.2. (2018).

[33] Hillebrand H, Dürselen C-D, Kirschtel D, Pollingher U, Zohary T (1999). Biovolume calculation for pelagic and benthic microalgae. J. Phycol..

[34] Neustupa J, Černá K, Šťastný J (2011). The effects of aperiodic desiccation on the diversity of benthic desmid assemblages in a lowland peat bog. Biodivers. Conserv..

[35] Schindelin J (2012). Fiji: an open-source platform for biological-image analysis. Nat. Methods.

[36] R Core Team. R: A Language and Environment for Statistical Computing. (2014).

[37] Osserman R (1978). The isoperimetric inequality. Bull. Am. Math. Soc..

[38] Klamkin MS (2006). Corrections to Ëlementary Approximations to the Area of N-Dimensional Ellipsoids. Am. Math. Mon..

[39] Warton DI, Duursma RA, Falster DS, Taskinen S (2012). smatr 3—An R package for estimation and inference about allometric lines. Methods Ecol. Evol..

[40] Revell LJ (2012). phytools: An R package for phylogenetic comparative biology (and other things). Methods Ecol. Evol..

[41] Škaloud P, Nemjová K, Veselá J, Černá K, Neustupa J (2011). A multilocus phylogeny of the desmid genus Micrasterias (Streptophyta): Evidence for the accelerated rate of morphological evolution in protists. Mol. Phylogenet. Evol..

