# Supplementary material for: Biological scaling in green algae: the role of cell size and geometry
Source: Sci Rep. 2021 Jul 13;11:14425. doi: 10.1038/s41598-021-93816-2 (PMC8277887; doi:10.1038/s41598-021-93816-2)
Supplement: Supplementary file 1 — Supplementary Information. [file 41598_2021_93816_MOESM1_ESM.pdf]

Scientific Reports

Supplementary information

Biological scaling in green algae: the role of cell size and geometry

Helena Bestová[1,2], Jules Segrestin[2], Klaus von Schwartzenberg[4], Pavel Škaloud[1], Thomas Lenormand[2], Cyrille Violle[2]

[1]Department of Botany, Faculty of Science, Charles University, Benátská 2, Prague, Czech Republic

[2]CEFE UMR 5175, CNRS – Université de Montpellier – Université Paul-Valéry Montpellier – EPHE, 1919 route de Mende, 34293 Montpellier, France

[4]Institute of Plant Science and Microbiology, Universität Hamburg, Ohnhorststr. 18, 22609 Hamburg, Germany

Corresponding author: Helena Bestová, helenabestova@gmail.com

This file contains two parts: *Appendix 1* with additional results and *Appendix 2* with details of surface and volume calculation.

Appendix 1

Table S 1 List of *Micrasterias* strains used in the study with measured growth rate and morphological traits. Strains originated form MZCH-SCVK Hamburg Collection (SCVK) and from CAUP Culture collection (K 605).

Taxon name		Strain number	Growth rate (day⁻¹)	Cell volume (µm³)	Cell surface (µm²)	Surface-to-volume	Cell mass (µg)	Mass-to-volume	α Circularity	β Degree of fractalization	γ Degree of flattening	δ The overall gain in surface
Micrasterias	*americana*	SCVK 126	0.106	121514.0	49373.6	0.41	1.46	12	0.113	3.15	1.33	4.19
Micrasterias	*apiculata*	SCVK 247	0.062	667273.8	251230.3	0.38	7.02	10.5	0.041	5.04	1.36	6.85
Micrasterias	*ceratofera*	SCVK 355	0.073	237339.3	82653.2	0.35	2.61	11	0.094	3.52	1.28	4.49
Micrasterias	*ceylanica*	SCVK 291	0.087	56042.4	18758.5	0.33	0.37	6.67	0.22	2.15	1.24	2.67
Micrasterias	*conferta*	SCVK 110	0.088	54565.4	29202.7	0.54	0.76	13.9	0.104	3.01	1.41	4.23
Micrasterias	*crux-melitensis*	SCVK 72	0.118	61617.8	24356.2	0.40	0.75	12.2	0.178	2.60	1.25	3.25
Micrasterias	*decemdentata*	SCVK 542	0.087	32087.4	13217.8	0.41	0.42	12.9	0.183	2.20	1.24	2.73
Micrasterias	*denticulata var. angulosa*	SCVK 50	0.043	730344.7	206832.5	0.28	5.24	7.17	0.064	3.84	1.38	5.31
Micrasterias	*fimbriata*	SCVK 41	0.064	743167.5	223232.4	0.30	8.89	12	0.072	4.15	1.37	5.67
Micrasterias	*furcata*	SCVK 75	0.095	55408.6	139748.4	2.52	1.66	30	0.026	11.75	1.70	20.03
Micrasterias	*jenneri*	SCVK 298	0.060	343337.3	65237.4	0.19	2.39	6.96	0.163	2.28	1.22	2.77
Micrasterias	*laticeps*	SCVK 121	0.058	235363.9	59206.9	0.25	2.20	9.35	0.156	2.64	1.23	3.24
Micrasterias	*muricata*	SCVK 125	0.078	85440.7	44866.7	0.53	1.29	15.1	0.105	3.95	1.22	4.82
Micrasterias	*novae-terrae*	SCVK 410	0.082	66836.5	65257.4	0.98	1.72	25.7	0.053	4.87	1.70	8.25
Micrasterias	*papillifera*	SCVK 71	0.060	199606.1	152294.2	0.76	1.85	9.27	0.035	5.34	1.74	9.29
Micrasterias	*pinnatifida*	SCVK 99	0.118	5669.4	7900.5	1.39	0.31	54.7	0.123	3.59	1.44	5.18
Micrasterias	*radians var. borogenesis*	SCVK 389	0.128	28709.0	31557.4	1.10	1.02	35.5	0.048	4.85	1.45	7.01
Micrasterias	*radiosa*	SCVK 154	0.138	64899.0	51905.9	0.80	0.69	10.6	0.053	4.74	1.41	6.70
Micrasterias	*ralfsii*	SCVK 300	0.080	114811.7	16549.6	0.14	3.32	28.9	0.488	1.32	1.11	1.46
Micrasterias	*rotata*	SCVK 26	0.066	585897.3	256928.2	0.44	2.07	3.53	0.057	4.75	1.61	7.64
Micrasterias	*thomasiana*	K 605	0.065	458193.0	256237.5	0.56	10.10	22	0.045	4.45	2.02	8.98
Micrasterias	*thomasiana*	SCVK 8	0.101	461782.5	221797.8	0.48	2.83	6.13	0.039	4.99	1.55	7.73
Micrasterias	*tropica*	SCVK 368	0.073	31095.2	12804.8	0.41	0.51	16.3	0.254	2.05	1.32	2.70
Micrasterias	*truncata*	SCVK 18	0.088	121432.9	28882.2	0.24	1.85	15.2	0.242	2.08	1.18	2.45

Table S 2 Results of phylogenetically informed reduced major axis regression of population growth rate and traits linked with body size. Parameter λ was estimated by ML.

		phylo RMA	
		slope	λ
Growth rate	*Cell mass*	-0.32	0.23
	Cell volume	-0.30	0.47
	Cell surface	-0.29	0.26
Cell mass	*Cell volume*	0.85	0.33
Cell surface	*Cell volume*	0.97	0.64
	Cell mass	1.12	0.42

Table S 3 Correlations among traits

	μ_{max}	*Body mass*	*Volume*	*Surface*	*Surface to volume*	*Density*	*Circularity*	*Flattening*	*Fractality*
Body mass	-0.52**								
Volume	-0.62**	0.79***							
Surface	-0.49*	0.76***	0.88***						
Surface-to-volume	0.40	-0.22	-0.34	-0.01					
Density	0.44*	-0.13	-0.44*	-0.33	0.61**				
Circularity	-0.02	-0.23	-0.35	-0.57**	-0.41*	0.10			
Flattening	-0.06	0.35	0.20	0.58**	0.51*	0.18	-0.65***		
Fractality	0.10	0.12	0.11	0.45*	0.84***	0.20	-0.65***	0.63***	
Surface gain	0.06	0.16	0.10	0.48*	0.83***	0.22	-0.63***	0.74***	0.98***

Values of Pearson's correlation coefficients and corresponding level of significance are displayed

(*$P < 0.05$, **$P < 0.01$, ***$P < 0.001$)

Table S 4 Correlations of morphological traits and residuals growth rate allometry scaling. Residuals come from mass-based and volume-based SMA regressions. None of the correlations was significant at 0.05 level

	residuals of			
	μ_{max} ~ *body mass*		μ_{max} ~ *cell volume*	
	Pearson's r	*p value*	*Pearson's r*	*p value*
Cell density	0.21	0.33	-0.29	0.17
Cell surface	0.29	0.17	0.36	0.12
Circularity	-0.21	0.32	-0.28	0.63
Degree of flattening	0.25	0.23	0.12	0.58
Degree of fractality	0.33	0.12	0.19	0.36

Scientific Reports

Appendix 2

Surface and volume calculations

This is a document showing calculation of surface and volume used in the manuscript. We create 3D model by dividing the shape into flat cross-sectional layers. Each layer is represented as frustrum a part of solid between two parallel planes. Originally the script is designed to calculate surface and volume of morphologically complicated cells of green algae Micrasterias. Here we show example of calculating surface and volume of sphere.

First, we take apical view of cell (in case of sphere - circle), divide it into specified number of crossectional layers and calculate length of each section.

Plot with example, where circle is divided into 10 layers per half of circle.

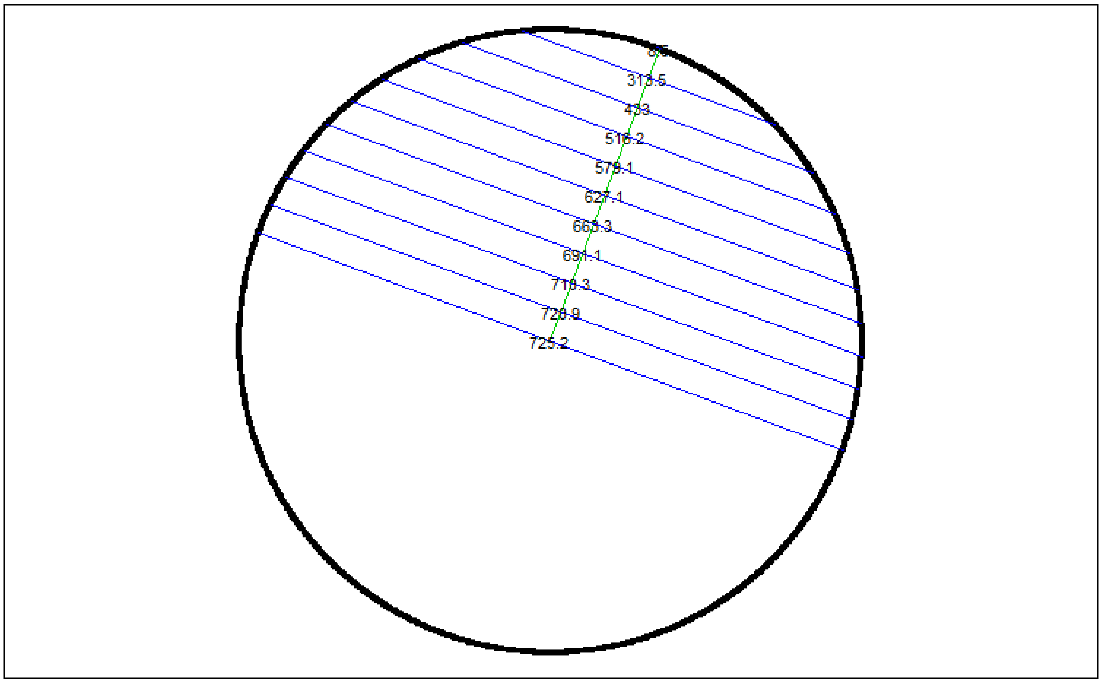

Second, we calculate volume and area of lateral side of each slide. This is done base on knowledge of area and perimeter of largest slice (frontal view of shape), width and thickness of slice.

```
frustrums.volume.surface  <- function(image, nsegments, quite = T) {
  volumes <- NULL
  surf.sides <- NULL

  #divides outline to slices and measure their length
  seg_length <- slices_length(image, nsegments, quite)
  #vector with length of slices
```

```
  seg_lengths <-  sort(seg_length$slice_length, decreasing = T)

  h <- seg_length$slice_thkness #thickness of slide
  f.area <- pi*(seg_lengths[1]/2)^2 #area of circle / frontal area
  f.perim <-  2* pi*(seg_lengths[1]/2) #perimetr of circle / frontal perimetr

  #for each slice calculate volume and surface of lateral side
  for(i in 1: (length(seg_lengths)-1 )) {

    #how much smaller is segment compare to diametr
    shrink.i <- seg_lengths [i] /seg_lengths [1]
    shrink.i1 <-  seg_lengths[i + 1] / seg_lengths [1]

    #volume of slice i, based on formula for frustrum
    vol.i <- h/3 * (f.area* shrink.i ^2 +  f.area * shrink.i1 ^2 +
                    (f.area* shrink.i ^2  * (f.area * shrink.i1 ^2)) ^ (1/2))
    volumes <- rbind(volumes, vol.i)

    #calculate slant height - distance measured on lateral side
    si <- (h^2 + ((seg_lengths [i] - seg_lengths [i+1])/2)^2) ^ (1/2)

    surf.side.i <- 1/2* si * (f.perim *shrink.i + f.perim *shrink.i1)
    surf.sides <- rbind(surf.sides, surf.side.i)}

  total_volume <- sum(volumes)*2
  total_surface <- 2*sum(surf.sides) + 2* f.area* ((seg_lengths [length(seg_l
engths)] / seg_lengths [1])^2)


  volume_sphere <- 4/3*pi*(seg_lengths[1]/2)^3
  surface_sphere <- 4*pi*(seg_lengths [1]/2)^2

  return(list(radius = seg_lengths[1]/2, frustrums_volume = total_volume,
              frustrums_surface = total_surface, sphere_volume = volume_spher
e,
              sphere_surface = surface_sphere, frustrum_SV = total_surface/to
tal_volume,
              sphere_SV = surface_sphere/volume_sphere))

}
```

We checked model if it converges towards correct value by running calculation on sphere of known diameter with number of slices varying from 1 to 100.

Red lines in plots represent actual surface and volume values

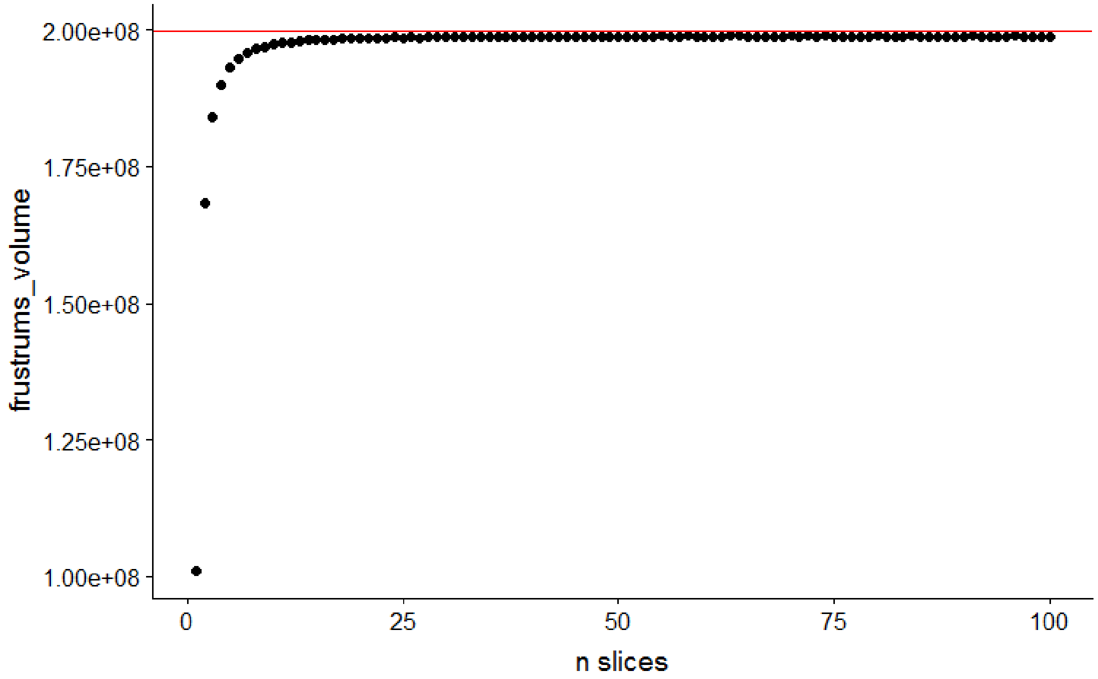

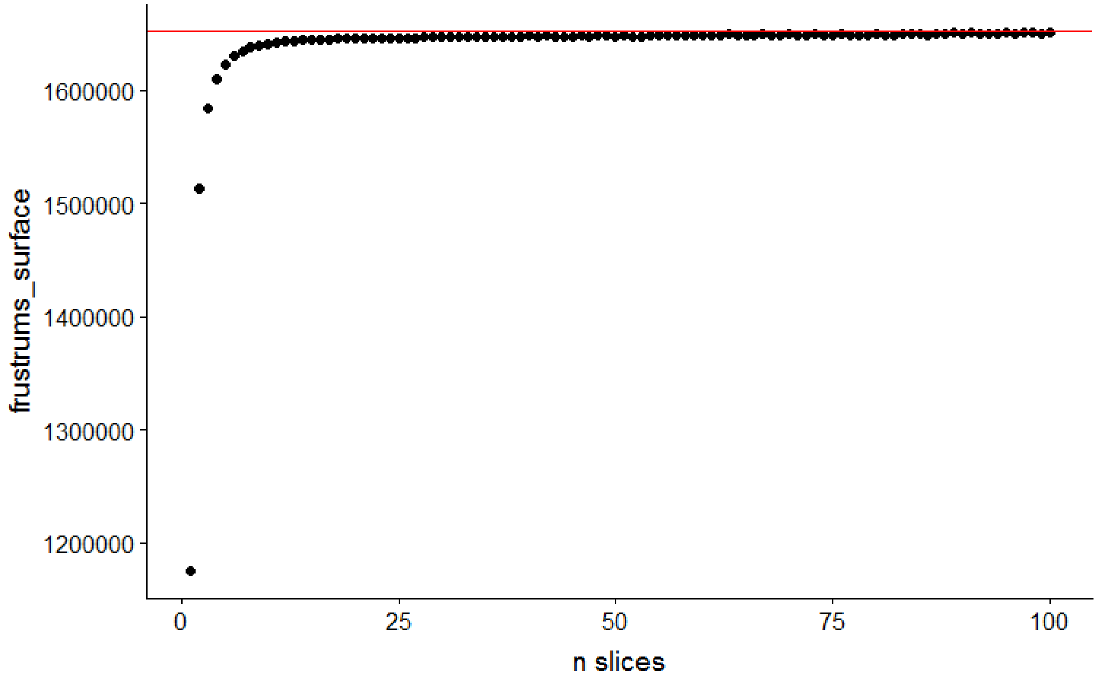

We ran this calculation on 4 spheres differing in size. The estimations of their surfaces and volumes are summarized in table below. Calculation of surface and volume was based on 100 slices per sphere (50 slides per hemisphere).

Estimated volumes and surfaces

	radius	frustrums_volume	frustrums_surface	sphere_volume	sphere_surface	frustrum_SV	sphere_SV
sphere 1	362.5962	198693915	1648019	199690927	1652176	0.0083	0.0083
sphere 2	615.6525	974305117	4760194	977452249	4763006	0.0049	0.0049
sphere 3	976.7344	3894013668	11971534	3903174788	11988443	0.0031	0.0031
sphere 4	1266.7905	8505897449	20156188	8515360426	20165987	0.0024	0.0024
